# Bloodletting Therapy for Patients with Chronic Urticaria: A Systematic Review and Meta-Analysis

**DOI:** 10.1155/2019/8650398

**Published:** 2019-04-16

**Authors:** Qin Yao, Xinyue Zhang, Yunnong Mu, Yajie Liu, Yu An, Baixiao Zhao

**Affiliations:** Beijing University of Chinese Medicine, Beijing 100029, China

## Abstract

**Background:**

Many trials have reported that bloodletting therapy is effective when treating chronic urticaria. There are currently no systematic reviews of bloodletting therapy for chronic urticaria.

**Objective:**

The aim of this review is to assess the effectiveness and safety of bloodletting therapy for chronic urticaria.

**Methods:**

A systematic review and meta-analysis of randomized controlled trials were performed. Disease activity control was assessed as the primary outcome. Response rate, recurrence rate, and adverse events were assessed as secondary outcomes.

**Results:**

Seven studies with 512 participants were included. One trial showed a significant difference between bloodletting therapy plus medicine and medicine alone in disease activity control (MD 0.67; 95% CI 0.03 to 1.31; p=0.04). Six trials (372 participants) showed a significant difference between bloodletting therapy and pharmacological medication in response rate (RR 1.10; 95% CI 0.97-1.26; P =0.15). Two studies (170 participants) showed a significant difference between bloodletting therapy plus pharmacological medication and pharmacological medication in response rate (RR 1.34; 95% CI 1.10-1.63; p=0.003). Two studies (126 participants) reported a statistically significant difference between bloodletting therapy and pharmacological medication in recurrence rate. No serious adverse events related to bloodletting therapy were reported.

**Conclusions:**

Bloodletting therapy might be an effective and safe treatment for chronic urticaria, but the evidence is scarce. More high quality trials are needed in the future.

## 1. Introduction

Chronic urticaria is a condition defined as the occurrence of wheals, angioedema, or both for more than 6 weeks [1]. The population prevalence estimate of chronic urticaria is 0.5-1%, and the risk of suffering from urticaria at least once during one's lifetime approaches 20% [2]. Individuals in all age groups suffer from chronic urticaria, especially between the ages of 20 and 40 [2]. The itching or physical discomfort during outbreaks of chronic urticaria negatively influences the patient's quality of life, affecting work and school performance, as well as sleep [1–3]. Patients' objective functioning and subjective well-being are both affected by chronic urticaria [4–6]. The disease also leads to a substantial economic burden to patients and society because of its high direct and indirect healthcare costs [4, 7, 8]. The aim of chronic urticaria management is complete symptom control, and reduction in urticaria symptoms and improvement in quality of life are recommended to assess the effectiveness of treatments [1]. The first-line pharmacological treatment for chronic urticaria is modern 2nd-generation H_1_-antihistamines [1, 9, 10].

Bloodletting therapy (BLT) is defined as the practice of treating diseases through the removal of a small amount of blood from patients [11]. Bloodletting therapy has been widely used around the world since the time of Hippocrates in the West and primitive society in China [12]. The instruments of bloodletting therapy include the triangle-edged needle, plum-blossom needle, injection needle, dermal needle, blades, vacuum blood sampling needle, etc., and cupping or leeches can often be used as an auxiliary method [13–15].

Bloodletting therapy originated thousands of years ago and has been used extensively in dermatosis [16–18]. Many clinical trials have reported the effect of bloodletting therapy in treating chronic urticaria in China [19–21]. There are currently no systematic reviews of bloodletting therapy for chronic urticaria. Therefore, we conducted this systematic review and meta-analysis to evaluate the effectiveness and safety of bloodletting therapy for patients with chronic urticaria.

## 2. Methods and Analysis

This review has been drafted under the guidance of the Cochrane Handbook for systematic reviews of interventions [22]. The review protocol was registered on PROSPERO (CRD 42018111143) and was also published on Medicine [23]. Consistency training for the review was conducted prior to beginning the review process.

### 2.1. Inclusion Criteria

Randomized controlled trials (RCTs) related to bloodletting therapy for treating chronic urticaria were included, without restrictions on language and publication status, while randomized crossover studies and quasi-randomized trials were excluded. Bloodletting therapy combined with a different type of complementary therapy (e.g., Chinese herb decoction, bloodletting therapy and other therapies) was excluded. The treatment comparisons consisted of bloodletting therapy compared with no treatment/placebo/sham bloodletting therapy/other active therapies or bloodletting therapy in addition to active therapy compared with the same active therapy. The primary outcome was disease activity control, measured by the urticaria activity score (UAS), urticaria control test (UCT), or other validated symptom scores [1]. The secondary outcomes included response rate, the recurrence rate during the follow-up period, quality of life, and adverse events.

### 2.2. Literature Search

The following databases were electronically searched without restriction to the publication status and language by two independent review authors (Yunnong Mu and Yajie Liu), from their inception to December 2018: the Cochrane Central Register of Controlled Trials (CENTRAL); PubMed; EMBASE; the Web of Science; Traditional Chinese Medicine databases; China National Knowledge Infrastructure (CNKI); Chinese Biomedical Literature Database (CBM); Chinese Scientific Journal Database (VIP database); and Wan-Fang Database. Reference lists of included studies and published reviews, conference proceedings, and unpublished literature were also searched as supplementary sources. Two trial registered platforms, Clinicaltrials.gov (http://www.clinicaltrials.gov) and the World Health Organization International clinical trials registry search portal (http://apps.who.int/trialsearch/), were searched for unpublished literature.

The search strategy used in PubMed was as follows: MeSH term “urticaria”; title/abstract: “chronic urticaria” or “hives” or “nettle-rash” or “angioedema” or “fong-tzen-kwai” or “wind-rash-patch” and MeSH term “bloodletting”; title/abstract: “bloodletting” or “phlebotomy” or “blood donation” or “collateral pricking therapy” or “blood-draining” or “leeching” and publication type “randomised controlled trial” or “controlled clinical trial”; title/abstract: “randomised” or “randomly” or “placebo” or “trial” or “groups”. Similar search strategies were conducted in all other databases.

### 2.3. Study Selection

EndNote software (V.X7) was used to remove duplicates and manage the studies. Two review authors (Yunnong Mu and Yajie Liu) independently screened the titles and abstracts of all retrieved studies to identify articles for full-text assessment and then independently scanned the full texts to confirm eligible trials. Disagreements were discussed by the two authors or arbitrated by the third author (Baixiao Zhao) when a consensus was not reached.

### 2.4. Data Extraction and Management

Two authors (Yu An and Xinyue Zhang) independently extracted data using a predesigned extraction form from included trials for the following information: general information, participants, methods, interventions, outcomes, results, adverse events, conflicts of interest, ethical approval, and other pieces of information.

### 2.5. Assessment of Risk of Bias in Included Studies

Two reviewers (Yu An and Xinyue Zhang) independently assessed the methodological quality for all included studies, with the Cochrane Collaboration's tool for risk of bias assessment. The following domains for risk of bias were assessed: sequence generation, allocation sequence concealment, blinding of participants, personnel and outcome assessors, incomplete outcome data, selective outcome reporting, and other sources of bias. The assessments were classified into three levels: low risk, high risk, and unclear risk. Any disagreements were discussed and arbitrated by the third author (Baixiao Zhao).

### 2.6. Measures of Treatment Effect

RevMan V.5.3 was used for data analysis and quantitative data synthesis. For continuous data, the standard mean difference (SMD) with 95% confidence intervals (Cis) was applied to measure the treatment effect. For dichotomous data, the risk ratio (RR) with 95% CIs was applied to measure the treatment effect.

### 2.7. Unit of Analysis Issues

Data from parallel-group studies were selected for analysis. In trials with multiple observation nodes, only data at the end of the treatment or the end of the follow-up were extracted for assessment. In studies with multiple groups, we split the “shared” group into two or more groups with smaller sample sizes and included two or more (reasonably independent) comparisons. In all studies, a single measurement for each outcome from each participant was collected and analyzed.

### 2.8. Missing Data

We tried to contact the first or corresponding authors of the included studies by email or telephone to get missing data. We only analyzed the available data when no additional data were obtained, with a discussion to judge the potential impact of the missing data.

### 2.9. Assessment of Heterogeneity

The Higgins I^2^ statistic was used to quantify heterogeneity among the included studies [22]. When the I^2^ value was less than 50%, significant heterogeneity was considered to be absent. When the I^2^ value exceeded 50%, statistic heterogeneity was considered to be present among the studies and the potential causes of the heterogeneity were explored.

### 2.10. Assessment of Reporting Biases

Funnel plots were used for the assessment of reporting biases and small-study effects. The plots were assessed visually or by Egger's test when 10 or more trial studies were included. Eligible trials were assessed via funnel plots regardless of their methodological quality.

### 2.11. Data Synthesis

RevMan V.5.3 statistical software was applied for data synthesis when a meta-analysis was allowed. The results were expressed as RR with 95% CI for dichotomous data and SMD with 95% CI for continuous data. If no significant heterogeneity existed, the fixed-effects model was used for data synthesis; otherwise, the random-effects model was conducted for data synthesis. We provided a systematic narrative synthesis to describe the characteristics and findings of the included trials if quantitative synthesis was not appropriate, such as insufficient RCTs or unidentified significant heterogeneity.

### 2.12. Subgroup Analysis and Sensitivity Analysis

There was no presubgroup plan. Subgroups of the different chronic urticaria types and bloodletting therapy methods were conducted when adequate data were obtained. Subgroup analysis or sensitivity analysis was considered when significant heterogeneity existed. The results were compared and discussed according to the pooled effect size.

### 2.13. Grading the Quality of Evidence

The Grading of Recommendations Assessment, Development and Evaluation (GRADE) working group methodology was applied to assess the quality of evidence for all outcomes [24]. The following domains were assessed: risk of bias, consistency, directness, precision, publication bias, and additional points. The assessments were graded into four levels: high, moderate, low, or very low.

## 3. Results

### 3.1. Study Selection and Study Characteristics

**Figure 1:**
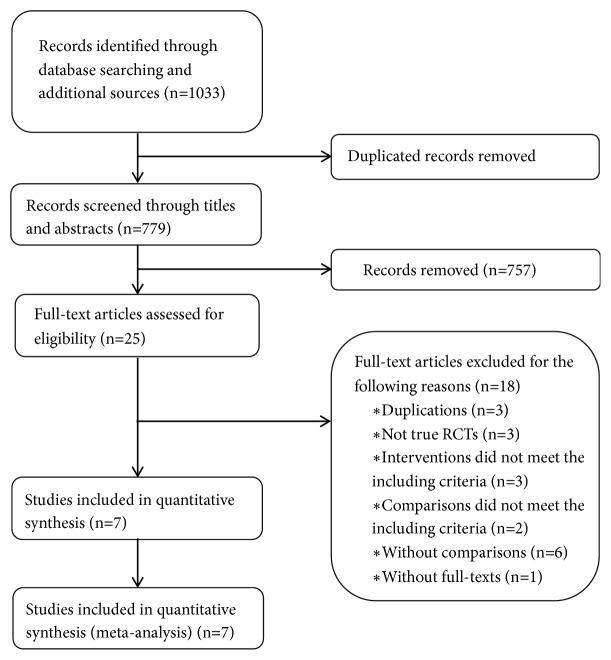
Flow diagram of the study selection process.

References (1033) were initially identified through electronic searching. Ultimately, 7 RCTs with 512 enrolled participants were included after stepwise screening [25–31]. The process of identifying trials is shown in Figure 1.

### 3.2. Characteristics of Included Trials

The characteristics of the included trials are summarized in Table 1. All 7 included trials were conducted in China and were published in Chinese between 2006 and 2018, and 1 of the 7 RCTs was an unpublished thesis for a master's degree [30]. The 7 trials were all single-center randomized controlled studies.

#### 3.2.1. Patients

A total of 512 participants in 7 trials were included, with sample sizes ranging from 54 to 110. The age of the patients ranged from 11 to 71 years. The disease course ranged from 6 weeks to 11 years. All included patients met the criteria for chronic urticaria. All included studies reported consistent baseline characteristics of sex, age, and disease duration. No additional information was obtained for missing data.

#### 3.2.2. Bloodletting Therapy Interventions

For the bloodletting tools, 5 RCTs used a triangle-edged needle combined with cupping, 1 RCT used a disposable syringe needle combined with cupping [25], and 1 RCT used a plum-blossom needle combined with cupping [29]. Acupoints at Du meridian and Bladder Meridian were applied the most in the included studies (5/7, 71.4%). Other acupoints used were Xuehai (SP 10), Quchi (LI 11), Zusanli (ST 36), Yuji (LU 10), and Chize (LU 5). The total number of treatments ranged from 4 to 30 times in 6 RCTs, while 1 RCT did not report the duration [26]. The most common frequency of interventions was every other day (5 trials; 71.4%), while the frequencies in the other 2 trials were once every 3 days (1 trial) or once a day (1 trial).

#### 3.2.3. Control Interventions

Two comparison patterns were contained in all 7 RCTs. Six of the 7 RCTs compared bloodletting therapy with pharmacological medications [25–30], and 2 of the 7 RCTs compared bloodletting therapy plus medication versus medication alone[30, 31]. It was noted that 1 of the 7 RCTs, containing 3 parallel arms, included a bloodletting therapy plus pharmacological medications arm, a bloodletting therapy arm, and a pharmacological medication arm, and this trial was analyzed in both comparison patterns [30]. Pharmacological medication included cetirizine, mizolastine, and loratadine, which are all 2nd-generation H1-antihistamines.

#### 3.2.4. Outcome Measures

The primary outcome (disease activity control) was reported in only 1 study. For the secondary outcomes (response rate; recurrence; adverse events; quality of life), all 7 studies reported response rate, 5 studies reported recurrence but only 2 reported it in an appropriate way, 5 studies reported treatment-related adverse events, and none of the trials reported quality of life.

### 3.3. Risk of Bias in Included RCTs

**Figure 2:**
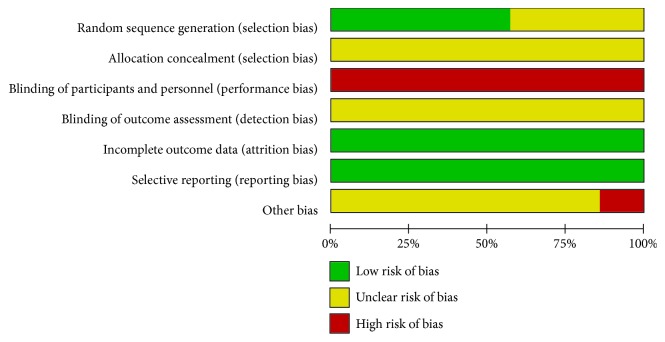
The risk of bias graph.

All included RCTs mentioned randomization. Four trials randomized using random number tables, and the other 3 trials did not mention specific randomization methods. The details of the allocation concealment were not reported in all 7 included studies, which resulted in an unclear risk. Due to the nature of bloodletting therapy, the blindness of bloodletting operators cannot be achieved. However, none of the included trials reported the blindness of the participants or outcome assessors. We graded all 7 studies as having a high risk in this domain. One trial reported 2 dropouts, where the missing outcome data were balanced in numbers and cause across intervention groups. The other 6 trials reported no withdrawals or dropouts. Thus, we found no attrition bias in all 7 studies. No trial registrations were searched, and no selective reporting was found in the studies. Thus, reporting bias was rated as unclear. The risk of bias assessment is presented in Figure 2. In Shi's study, the total treatment time and the outcome evaluation time point were not clearly reported; thus, we rated the other bias of this study as high.

### 3.4. Synthesis of Results

Seven trials were divided into two parts to conduct meta-analysis based on the different types of comparison groups. Then, trials with similarities were pooled together. Subgroup analysis was not conducted, as there were an insufficient number of studies included in this review.

#### 3.4.1. Bloodletting Therapy versus Pharmacological Medication

*Disease Activity Control*. Only Li's study reported disease activity control using a 0-18 score scale [27]. They only reported the disease activity control score at baseline and after treatment. The disease activity control score change from baseline to after treatment was calculated using the formula recommended by the Cochrane Handbook [22].

**Figure 3:**
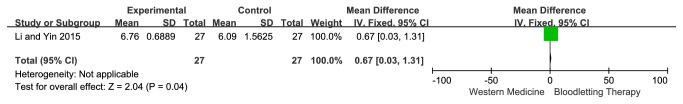
Forest plot of comparison: bloodletting therapy versus pharmacological medication; outcome: disease activity control.

The MD was 0.67 (95% CI 0.03-1.31, p=0.04) using the fixed model (Figure 3). There was a statistically significant difference between bloodletting therapy and pharmacological medication (cetirizine) in disease activity control. GRADE analysis indicated that the overall quality of the evidence for this outcome was very low due to a high risk of bias and the imprecision and sparseness of the data. 

**Figure 4:**
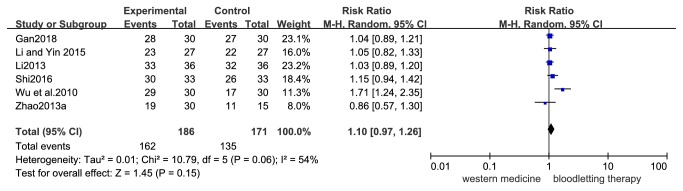
Forest plot of comparison: bloodletting therapy versus pharmacological medication; outcome: response rate.

*Response Rate*. Six trials (357 participants) compared the effects of bloodletting therapy versus medication (loratadine, cetirizine, or mizolastine). There was no statistically significant difference between bloodletting therapy and pharmacological medication in the response rate. The RR for the response rate was 1.10 (95% CI 0.97-1.26; P =0.15; I^2^ = 54%, Figure 4). GRADE analysis indicated that the overall quality of the evidence for this outcome was very low due to a high risk of bias and the imprecision, inconsistency, and sparseness of the data. 

*Recurrence Rate*. Two trials (126 participants) reported the recurrence rate in a proper manner [25, 26]. Shi reported that the bloodletting therapy group showed a lower recurrence rate than cetirizine (P<0.05), without mention of the follow-up time [26]. Gan reported that the bloodletting therapy group showed a lower recurrence rate than cetirizine over 1 month of follow-up (P<0.05) [25].

#### 3.4.2. Bloodletting Therapy plus Pharmacological Medication versus Pharmacological Medication

**Figure 5:**
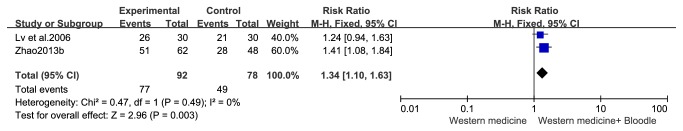
Forest plot of comparison: bloodletting therapy plus pharmacological medicine versus pharmacological medication; outcome: response rate.

*Response Rate*. Two studies (170 participants) compared bloodletting therapy plus pharmacological medication versus the same medication with regard to response rate [30, 31]. The RR for the response rate was 1.34 (95% CI 1.10-1.63; p=0.003; I^2^ = 0%, Figure 5). There was a statistically significant difference between bloodletting therapy plus pharmacological medication and pharmacological medication regarding the response rate. GRADE analysis indicated that the overall quality of the evidence for this outcome was low due to a high risk of bias and the imprecision and sparseness of the data.

### 3.5. Adverse Events

No serious adverse events were reported in all 7 included trials. Two studies did not mention adverse events at all [26, 29]. One study reported that no adverse events occurred [30]. For bloodletting therapy, 2 cases of hematoma were reported in Gan's study, but the hematoma was eliminated after ironing with Chinese herbal medicine [25]. For pharmacological medication treatment, 4 studies reported 14 cases of dry mouth, 5 cases of headache, 25 cases of drowsiness, 1 case of vomiting, 1 case of dizziness, and 5 cases of fatigue [25, 27, 28, 31]. For bloodletting therapy plus pharmacological medication treatment, 1 study reported 1 case of drowsiness and 1 case of dry mouth [31].

## 4. Discussion

### 4.1. Summary of the Main Findings

Despite an extensive literature search, only 7 studies with 512 participants were included and synthesized in this meta-analysis. The result of the meta-analysis indicated that, compared with pharmacological medication, bloodletting therapy seemed to be more effective at improving disease activity control (MD 0.67, 95% CI 0.03 to 1.31) and had no difference in response rate (RR 1.10, 95% CI 0.97-1.26), which indicated that bloodletting therapy might have a prior effect on disease activity control and an equal effect compared to pharmacological medication. Data from two studies showed that bloodletting as an adjuvant therapy enhanced the effect of pharmacological medication on the response rate (RR 1.34, 95% CI 1.10-1.63). For the outcome of recurrence rate, 5 studies reported a number of recurrent participants. However, the data acquisitions were improper in 3 studies, and the included total number of participants is incomplete. The results of the 2 included trials (126 participants) indicated that bloodletting therapy might have a better long-term effect for the treatment of chronic urticaria compared to pharmacological medication. Quality of life was not reported in all the included studies. For the safety evaluation, no serious adverse reactions were reported to be associated with bloodletting therapy, and hematoma might be the only potential adverse reaction.

### 4.2. Applicability of Evidence

As there were many methodological defects in the included studies, we must be careful in explaining the results. The processes of randomization generation were not clear in 2 of the included studies, and the allocation concealment was not mentioned in all 7 included studies, which led to an unclear selection bias. None of the studies applied participant and personnel blinding, which led to a high performance bias, and there was no report on assessment blinding, which led to an unclear detection bias. None of the included studies were registered in a clinical trials registry platform, which led to an unclear reporting bias. In addition, all included studies were conducted in China, which limited the population to which the conclusion may be applicable.

Cetirizine, mizolastine, and loratadine are all 2nd-generation H_1_-antihistamines and are considered effective medicines recommended by EAACI/GA^2^LEN/EDF/WAO guidelines. The results demonstrated that bloodletting therapy seemed to have equal effects as these medicines in response rate and might be more effective at disease activity control, which indicated that bloodletting therapy has potential clinical application value. The results that bloodletting plus these medicines as an adjuvant therapy seemed to be more effective than the medicines alone indicate that bloodletting therapy could act as a possible adjunct therapy to pharmacological medication when treating chronic urticaria. However, the small sample size without sample size calculation and the unclear design of the included studies, such as superiority test and noninferiority test, make it difficult to ensure whether there was enough power to detect the between-group difference. There were no studies comparing bloodletting therapy to no intervention or placebo/sham bloodletting therapy, so the specific treatment effect of bloodletting therapy for chronic urticaria was not clear.

### 4.3. Limitations of This Review

There are some limitations to this review, and the results should be interpreted cautiously. First, the sample size of included studies is small. Second, only Chinese and English databases were searched, which will probably lead to the exclusion of some relevant studies published in other languages. Third, the combination of different area selection and duration types of bloodletting therapy may cause significant clinical heterogeneity. A study about how to achieve the most effective bloodletting therapy may also need to be conducted in the future. Besides, the Global Allergy and Asthma European Network (GA^2^LEN) recommended patient-reported outcomes (PROs) and health-related quality of life in patients with urticarial [32, 33]. PROs have been recommended to be reported for randomized controlled trials [34]. The included trials were all published in Chinese and all used comprehensive outcomes, such as response rate, as primary outcomes, lacking for universal, and patient-reported outcomes. The comprehensive outcomes, which combine the clinical symptoms, signs, and laboratory examinations as one outcome, are not internationally recognized and cannot reflect the characteristics of interventions. Using comprehensive outcomes is also the common problems of most randomized controlled trials of traditional Chinese medicine published in Chinese [35, 36].

### 4.4. Implications for Practice and Research

Though the data showed potential effectiveness of bloodletting therapy in chronic urticaria, the quality of the evidence is low in this review, and there were many aspects that can be improved in future studies. For the study design, it is important to choose an appropriate experimental design for different study purposes. For the methodological quality, the included studies were poor, including registration, sample size calculation, the processes of randomization and allocation concealment, and blinding. Registration should be implemented for every study, and quality control should be conducted throughout the entire study process in the future. As for outcome measures, patient-reported outcomes and universal measures should be used, such as the urticaria activity score (UAS) and urticaria control test (UCT) recommended by EAACI/GA^2^LEN/EDF/WAO guidelines, which has been widely used and verified [1, 37–39]. In addition, future studies should pay more attention to quality of life and follow-up assessment. Therefore, to provide convincing proof, large-scale multicenter RCTs with proper outcome measurements and long-term follow-up are recommended.

## 5. Conclusion 

In conclusion, bloodletting therapy may potentially be effective for disease activity control in chronic urticaria, with a very low degree of quality of the evidence. Bloodletting therapy might be safe for treating patients with chronic urticaria, according to the current limited evidence. For future research, large-scale multicenter RCTs with proper outcome measurements and long-term follow-up should be conducted to provide convincing proof.

## Figures and Tables

**Table 1:** The characteristics of included trials.

Reference	Design	Comparisons	Bloodletting regimen	Treatment frequency and duration	Outcomes
Gan 2018 [25]	Parallel, 2 arms	bloodletting, 30 cetirizine, 30	disposable syringe needle with cupping; Geshu (BL 17), Feishu (BL 13), Pishu (BL 20)	every other day; a total of 4 times	response rate; recurrence rate; adverse events

Shi 2016 [26]	Parallel, 2 arms	bloodletting, 33 cetirizine, 33	triangle-edged needle with cupping; Feishu (BL 13), Pishu (BL 20), Quchi (LI 11), Dazhui (DU 14), Xuehai (SP 10), Zusanli (ST 36)	every other day; duration not mentioned	response rate; recurrence rate

Li and Yin 2015 [27]	Parallel, 2 arms	bloodletting, 27 cetirizine, 27	triangle-edged needle with cupping; Yuji (LU 10), Dazhui (DU 14), Weizhong (BL 40), Chize (LU 5)	every other day; a total of 4 weeks	response rate; disease activity control; recurrence rate; adverse events

Li 2013 [28]	Parallel, 2 arms	bloodletting, 36 loratadine, 36	triangle-edged needle with cupping; Xuehai (SP 10), Quchi (LI 11), Weizhong (BL 40)	every 3 days; 3 times for a course, a total of 2 courses	response rate; adverse events

Wu et al. 2010 [29]	Parallel, 2 arms	bloodletting, 30 cetirizine, 30	plum-blossom needle with cupping; Du meridian and Bladder Meridian	every other day; 10 times a course, a total of 2 courses, 5 days interval between courses	response rate

Zhao 2013 [30] unpublished thesis	Parallel, 3 arms	bloodletting, 30 mizolastine, 30 bloodletting plus mizolastine, 30	triangle-edged needle with cupping; Dazhui (DU 14), Feishu (BL 13), Geshu (BL 17), Ganshu (BL 18), Pishu (BL 20)	every other day; 4 weeks	response rate; recurrence rate; adverse events

Lv et al. 2006 [31]	Parallel, 2 arms	bloodletting plus cetirizine, 62 cetirizine, 48	triangle-edged needle with cupping; Dazhui (DU 14), Feishu (BL 13), Dachangshu (BL 25), Pishu (BL 20), Shenshu (BL 23)	once a days for consecutive 4 days as a course; 3 days interval between courses; a total of 4 weeks	response rate; recurrence rate; adverse events
